# Local Anesthesia in Surgery of the Colon and Rectum

**Published:** 1917-04

**Authors:** Wm. M. Beach


### Local Anesthesia in Surgery of the Colon and Rectum.

BY WM. M. BEACH.

Beach's conclusions fully detailed in the *International Clinics* for March on the subject are as follows:

1. Eliminating terrorism associated with operations under general anesthesia.

2. Absence of post-operative distress and complications.

3. The anesthesia is complete, thoroughly blocking the field, thus preventing shock.

4. It persuades the patient to undergo an operation because the detention from business is shorter and post-operative pain is less.

5. Skill in technic is achieved by virtue of the surgeon's care in gendle handling of a conscious patient.

6. It will teach him to handle tissues more deftly in general anesthesia, realizing that much pain and tendency to infection follows tearing and mutilating of soft parts.

7. Local anesthesia conserves the patient's peace of mind, as there are many who will testify to its efficiency and complete relief with so little inconvenience.

